# An Influence of the Law

**Published:** 1898-06

**Authors:** 


### An Influence of the Law.

At the last meeting of the Academy of Medicine of Cincinnati, Dr. C. A. L. Reed made a report of the workings of the Ohio medical law, that was of very great interest to all physicians. He showed that the work of registration alone caused a disappearance from the medical profession of nine hundred and sixty unqualified physicians. These men did not all leave the State, but they did quit practicing medicine; most of them entirely disappeared. Seven thousand four hundred were qualified to register, showing about one in eight to have been unqualified, which is an enormous percentage.

In Hamiton County, with nearly eight hundred practitioners, eighty-four were found to be disqualified, of whom more than fifty have disappeared, twenty-four are under indictment, and the county prosecutor begs the board to hold up with its prosecutions because of his crowded docket.

This showing is splendid, and indicates that all statements of the large number of unqualified men in Ohio were not exaggerated. The writer does not remember to have ever heard that one-tenth of all were unqualified; the showing of one in eight is really appalling. The law was enacted none too soon, and its enforcement has been reasonably vigorous.

Another satisfactory statement made by Dr. Reed, was a declaring by the Supreme Court of the State that every part of the law is constitutional.

The board has issued an official register of the physicians of the State that is of great utility.—*The Cincinnati Lancet-Clinic.*

